# An Interrupted Time Series Analysis of the Impact of the COVID-19 Pandemic on Routine Vaccination Uptake in Kenya

**DOI:** 10.3390/vaccines12080826

**Published:** 2024-07-23

**Authors:** Michael Ngigi, Yola Moride, Anne-Marie Castilloux, Sue Ann Costa Clemens

**Affiliations:** 1 Institute for Global Health, Centro Servizi di Ateneo Santa Chiara Lab, University of Siena, 53100 Siena, Italy; sue.costa@unisi.it; 2 Department of Health Services, Kericho County, P.O. Box 112, Kericho 20200, Kenya; 3 Center for Pharmacoepidemiology and Treatment Science, Rutgers, The State University of New Jersey, New Brunswick, NJ 08854, USA; morideyo@ifh.rutgers.edu; 4 Yola RX Consultants, Montreal, QC H3H 1V4, Canada; 5 Oxford Vaccine Group, Department of Paediatrics, University of Oxford, Oxford OX3 7LE, UK

**Keywords:** public health, epidemiology, immunization, vaccination, interrupted time series, COVID-19, routine immunization, modelling, ARIMA

## Abstract

A strategic priority of the World Health Organization’s Immunization Agenda 2030 is to increase vaccination coverage and equity through reaching “zero-dose” children. Through an ecological study, we sought to quantify the impact of the COVID-19 pandemic on the coverage of the pentavalent and the measles/rubella vaccines in Kenya, without implying causality. The monthly number of doses from January 2017 to August 2022 were obtained from the Kenya Health Information System for the pentavalent and the measles/rubella vaccines. Immediate (step) and long-term (ramp) changes following interruptions occurring during the period from March 2020 to December 2020 were assessed through an interrupted time series analysis using an autoregressive integrated moving average (ARIMA) model, accounting for seasonality. In December 2020, there was an immediate decrease of 8337, 12,212, and 20,848 in the number of doses for the first, second, and third dose of the pentavalent vaccine, respectively (statistically significant for the third dose only). This corresponded to a percentage relative difference of −21.6, −20.1, and −24.5, respectively, for three doses of pentavalent vaccines, while for measles/rubella vaccine it was −27.3 and −33.6, respectively, for the first and second dose. COVID-19 resulted in interruptions affecting routine immunization, but recovery occurred within four months.

## 1. Introduction

As vaccine-preventable diseases are a leading cause of death and disability worldwide, vaccines are one of the most effective interventions for global health. Based on modelling from the World Health Organization (WHO)/UNICEF Estimates of National Immunization Coverage (WUENIC), there were 29.7 million deaths averted by vaccination between 2001 and 2010 [1]. A modelling study estimated that 51 million deaths will be averted due to vaccinations between 2021 and 2030 [1]. The majority of the averted deaths (18.8 million) would be due to measles, followed by 14 million deaths due to hepatitis B, and 4.1 million deaths due to human papillomavirus (HPV). Notably, half of the averted deaths will occur in low- and middle-income countries, with 23% in the WHO Regional Office for Africa (AFRO) region, while only 22% and 4% would occur in middle- and high-income countries, respectively [1].

One of the strategic priorities of the WHO Immunization Agenda 2030 is to increase coverage and equity for vaccination. This will be achieved through extending immunization services to reach “zero-dose” children, as well as getting and sustaining high immunization coverage in nations and districts globally [2]. The United States Centers for Disease Control and Prevention (CDC) defines vaccine coverage as the estimated percentage of people who have received specific vaccines [3], which is an important indicator of how populations are protected against vaccine-preventable diseases. Additionally, it enables areas with low coverage to be identified to ramp up immunization campaigns in those communities.

External factors can affect vaccine coverage, such as the cost of vaccines, access to health care, and beliefs about vaccines. In order to increase vaccine coverage, it is important to address these factors [4]. For instance, many low- and middle-income countries struggle to afford vaccines, so WHO provides financial assistance through Gavi, the Vaccine Alliance, for vaccine procurement and supply. It is also important to educate people about the importance of vaccines. Misinformation about vaccines is common and can lead to people making decisions that put themselves and others at risk. For example, the anti-vaccine movement has led to a decline in vaccination rates in some countries, and has been blamed for a number of outbreaks, such as the 2015–2016 measles outbreak in the United States [5,6,7]. Addressing vaccine hesitancy is essential to maintaining high vaccination rates. Health care providers play a key role in this, as they are often the first point of contact for people with questions about vaccines. It is important for providers to be up to date on the latest information about vaccines, and to be able to effectively communicate this information to their patients. Vaccines are a vital part of protecting people from diseases, and ensuring high vaccination rates is essential to maintaining population health [8,9,10].

To measure access, Gavi, the Vaccine Alliance, defines zero-dose children as those who have not received the first dose of the diphtheria-tetanus-pertussis (DTP)-containing vaccine (DTP1) [11]. Furthermore, an under-immunized child is defined as a child missing the third dose of DTP (DPT3). A fully immunized child is defined as an infant who has received the Bacillus Calmette–Guerin (BCG) vaccination, three doses of a DTP vaccine, three doses of the polio vaccine, and one dose of a measles vaccine, all within their first year of life [12,13].

The COVID-19 pandemic that began in 2019 has impacted the uptake of routine childhood vaccinations all over the world [14]. The WHO reported that DTP3 coverage decreased by 3% from 2019 to 2020; likewise, measles vaccine coverage dropped by 2% globally to levels last seen in 2014, with the WHO South East Asia and African region dropping by 6% and 2%, respectively, between 2019 and 2020 [15]. Globally, these falls in coverage translate into 3.7 million more children not being vaccinated with DTP3 in 2020 (19.0 million) compared with 2019 (22.7 million). About 75% of those who did not receive DTP3 in 2020 were zero-dose children [16]. Surveys carried out during the pandemic indicate that reduced coverage in vaccines was due to disruptions to routine immunization services occasioned by reduced supply in vaccines and vaccine-related supplies, travel restrictions, lower access to healthcare workers, and challenges among healthcare workers in accessing personal protective equipment [17].

In Kenya, disruptions to vaccination were caused by public health interventions implemented at the height of the pandemic to slow down its spread. These included controlled lockdowns, physical distancing in public transport and public areas, dusk to dawn curfews, and school closures [18,19]. In addition, healthcare worker strikes occurred in Kenya during the pandemic period [20].

Globally, the proportion of one-year-olds immunized with DPT3 is an important proxy indicator for the strength of the public health system to provide essential health services [14,21]. On the other hand, the proportion of children receiving the first dose of the measles vaccine is an important coverage indicator as it correlates with the number of fully immunized children [22].

Time series analysis is frequently used to describe trends over time in drug (or vaccine) usage or health outcomes in the population [23,24,25,26,27,28]. Time series may be defined as a sequence of observations taken periodically over time. In public health, this could, for instance, be the recorded daily number of persons immunized with a certain vaccine, the weekly number of reported cases of an infectious disease, or the monthly number of patients receiving treatment with antibiotics. Since adjacent observations are usually, but not always, dependent, time series data can be used to predict future observations based on previous observations [29].

Interrupted time series (ITS) analysis has been used as a quasi-experimental design to assess the impact of an intervention where randomized controlled trials are not feasible [26,30]. Notably, in public health, it has been implemented to assess the effect of various interventions such as drug policy changes, guideline changes, and new vaccinations [24,26,27]. While the COVID-19 pandemic cannot be considered as an intervention, this method is particularly adapted to assess the impact of an event that has occurred at the country level at one point in time in the population (e.g., a pandemic), given that there were no “unexposed” controls, i.e., populations unexposed to the pandemic.

Various approaches have been used to implement ITS in the literature. These include segmented regression, linear regression, segmented linear regression [26], autoregressive integrated moving average models (ARIMA) [31], and a mixture of more advanced models such as logistic regression and Poisson models. ARIMA and seasonal ARIMA (SARIMA) have been implemented widely in the analysis of time series data. For instance, Vanella et al. combined principal component analysis with SARIMA analysis to develop the Lee–Carter Index and used it to assess excess mortality due to COVID-19 in 19 countries [32,33]. Other implementations of ARIMA modelling with newer methods such as PROPHET have been implemented to assess daily new cases of COVID-19 [34]. In this study, we opted to use ITS with ARIMA modelling.

This research sought to quantify, without implying causality, the impact of COVID-19 pandemic on coverage of two childhood vaccines included in the routine immunization program in Kenya—the diphtheria, pertussis, tetanus, hepatitis B, and Hemophilus influenzae type b DPT-HepB-Hib (pentavalent) vaccine (administered within the first six months of life), and the measles/rubella vaccine (targeted to 9-month-old children)—to inform future campaigns in targeted areas.

## 2. Materials and Methods

### 2.1. Source of Data

Data were obtained from the Kenya Health Information System (KHIS), a district health information system 2 (DHIS2)-based system [35] deployed and maintained by the Kenya Ministry of Health for the reporting of health data. Data are collated monthly by facility in charges at facility level from the primary registers and documents, and are filled in physical reports. These reports are then submitted to the subcounty, where they are validated and then entered to the KHIS. The data obtained contained coverage and uptake data of the pentavalent and measles–rubella vaccine, administered between 6 weeks to 2 years of life in infants in Kenya.

### 2.2. Significant Public Health Events in Kenya before and during the COVID-19 Pandemic

Before the COVID-19 pandemic, a significant doctor’s strike took place between 5 December 2016 to 14 March 2017, lasting a total of 100 days [36,37]. This was followed by a 150-day nurses’ strike which took place between 5 June 2017 to 3 November 2017 [38,39].

The first case of coronavirus in Kenya was confirmed on 13 March 2020, marking the beginning of the COVID-19 pandemic in Kenya [40]. On 20 March 2020, all schools and institutions of higher learning were closed down to stem the spread of COVID-19 [41]. A nationwide curfew for all except essential personnel was subsequently instituted five days later on 25 March 2020, which was to last between 7 pm in the evening and 5 am in the morning. This was adjusted two days later to a nationwide dusk to dawn curfew on 27 March 2020 [42,43].

On 6 April 2020, there was a restriction of all but essential movement out of and into the capital city county of Nairobi [44]. Two days later on 8 April 2020, a similar order was placed for the counties of Mombasa, Kwale and Kilifi which were seeing a rising number of cases [45]. Mandera county joined the fray on 22 April 2020 for similar reasons [46]. The measures were heightened in May 2020 with cessation of movement out of specific areas of Nairobi and Mombasa Cities and closure of markets, restaurants and eateries within those areas which lasted until early June of the same year [47].

On 17 June 2020, the Government of Kenya announced further restrictions in response to the worsening of the COVID-19 pandemic. Restrictions were focused on 13 counties declared “hotspot zones”, specifically Busia, Vihiga, Kisii, Nyamira, Kakamega, Trans Nzoia, Bungoma, Kericho, Bomet, Siaya, Kisumu, Homa Bay, and Migori. The restrictions included suspension of public gatherings and an adjusted curfew to run from 7 pm to 4 am, while other counties maintained the existing curfew hours of 10 pm to 4 am and the restriction of movement between the hotspot zones and other areas of the country, except for essential and emergency services. Non-food and livestock markets were also suspended for a period of 30 days, employers were strongly advised to allow employees to work from home, all physical and worship gatherings were also suspended for a duration of 30 days, hospitals were to limit visitors to one individual per patient, and funerals were to be held within 72 h of death confirmation with attendance limited to 50 people, while weddings were restricted to a maximum of 30 guests [48].

As the first wave of COVID-19 eased, the measures were relaxed. On 7 July 2020, cessation of movement imposed on the Nairobi, Mandera, Kilifi, Kwale, and Mandera Counties was lifted [49].

A major healthcare worker strike comprising nurses and clinical officers occurred between 7 December 2020 and 23 February 2021. The healthcare workers wanted more risk allowances, personal protective equipment, and priority in vaccination [20].

The curfew was eventually lifted on 20 October 2021, and on 11 March 2022, the government lifted all remaining COVID-19 restrictions, including a ban on large indoor gatherings such as religious services and the requirement to present a negative COVID-19 test for arriving air passengers [49,50].

The political campaign season began on 29 May 2022, lasting until 6 August 2022 [51,52]. The general election in Kenya was held on 9 August 2022 [53].

### 2.3. Statistical Analysis

National monthly data on immunization, aggregated from facility data, were obtained from the Kenya Health Information System from January 2017 to August 2022. Available data on counties started in January 2018. Interrupted autoregressive integrated moving average (ARIMA) models to examine the association of the pandemic with patterns of monthly vaccination were conducted over this period. The ARIMA models allows for an examination of changes in vaccination while accounting for autocorrelation between consecutive observations (i.e., autocorrelation) and seasonality [26]. The pre-pandemic period covered 38 months (January 2017 to February 2020), while the pandemic period covered 30 months (March 2020 to August 2022). Models with a step intervention function (to assess the immediate effect of an interruption) and a ramp intervention function (for the long-term effect) were used [54].

Interruptions were identified visually from graphs and selected from a list of publicly available public health measures or notable events that occurred after the start of the COVID-19 pandemic in Kenya in March 2020 (see Appendix A).

The monthly vaccination uptake data represent realizations of a stochastic process which yield a time series that can be denoted by $Z_{t}$, which represents a time series with terms $Y_{t}$, $Y_{t+1}$……. $Y_{t+n}$, and *t* represents a timepoint in the time series.

The basic ARIMA model for $Y_{t}$, the tth observation of the time series of a specific vaccine can be represented as the following:

(1) $$Y_{t}=N\left(a_{t}\right)+X\left(I_{t}\right)$$

In which monthly uptake of a vaccine $Y_{t}$ is represented as a sum of a ‘noise’ portion $N(a_{t})$ and a portion representing the intervention $X(I_{t})$. $N(a_{t})$ is a non-linear function of $a_{t}$, which is the tth observation of a time series with white noise property, such as an outcome of a random process which is normally distributed with a mean of 0 and variance of $\sigma^{2}$ [55].

$$a_{t\;}\sim iid\;N(0,\;\sigma_{a}^{2})$$

The $N(a_{t})$ component was modelled as a seasonal ARIMA process, represented as *ARIMA*(*p*, *d*, *q*)(*P*,*D*,*Q*)*_s_*. Where *p* is the autoregressive order, *d* is the differencing order, *q* refers to the moving average term of the nonseasonal part of the model, while *P*, *D*, and *Q* have the same role for the seasonal part of the model, and s indicates the number of seasons.

Similarly, $X(I_{t})$ is a nonlinear function of $I_{t}$, $I_{t}$is the tth observation of a binary variable $I_{t}$, which represents the presence or absence of the intervention [55]. This was modelled additively using the step function to assess abrupt and persistent changes, and using the ramp function to assess gradual recovery.

For the step function, it was hypothesized that the pandemic caused a sudden downward shift in vaccination uptake, and the step variable at time *T* takes the value 0 before the start of the intervention (*I*) and 1 afterwards, as shown below.

$$I_{t}=\left\{\begin{array}{c} 0,\;if\;t<I\\ 1,\;if\;t\geq I \end{array}\right.$$

This impacted $Y_{t}\;$through the first order transfer function $X(I_{t})$ as the following:

(2) $$X\left(I_{t}\right)=\omega_{0}I_{t}\;$$

where $\omega_{0}$ represents the initial value for the impact of the intervention at the time of the intervention (*I*).

For the ramp function, it was hypothesized that the vaccination uptake rose after the initial drop in vaccination uptake, and the ramp variable takes the value of 0 immediately before the intervention (*I*) and increases by 1 afterwards, as shown below.

$$I_{t}=\left\{\begin{array}{l} 0,\;if\;\;\;\;\;\;t<I\\ t-I+1,\;if\;t\geq I \end{array}\right.$$

This impacted $Y_{t}$through the second order transfer function $X(I_{t})$ as;

(3) $$X\left(I_{t}\right)=\frac{\omega_{0}}{1-\delta_{1}B}I_{t}\;$$

where $\delta_{1}$ is the rate of change post intervention and Β is the backshift operator which shifts the time series backwards (i.e., $ΒY_{t}=Y_{t-1}$).

Therefore, the combined model was the following:

$$Y_{t}=N\left(a_{t}\right)+\omega_{0}I_{t}+\frac{\omega_{0}}{1-\delta_{1}B}I_{t}\;$$

Which simplifies to the following:

(4) $$Y_{t}=N\left(a_{t}\right)+\left(\omega_{0}+\frac{\omega_{0}}{1-\delta_{1}B}\right)I_{t}\;$$

Selection of the ARIMA terms was conducted by the auto.Arima function of the R statistical package using an algorithm proposed by Hyndman et al. [56]. The fit of the model was examined using autocorrelation function (ACF) and partial autocorrelation function (PACF) plots, normality plots of the model residuals, and Ljung–Box tests for stationarity. The ACF and PACF plots confirmed the autoregressive, moving average, and differencing orders, while normality plots for the model residuals confirmed that the residuals were normally distributed and had a white noise property [31].

The selected ARIMA models were then fit to the pre-interruption data, and monthly vaccine uptake were forecasted for the period after the interruption as if no interruption had occurred (the counterfactual). The actual observed values post-interruption were assessed against the model forecast, with both the step and ramp functions to assess whether statistically significant differences in their mean had occurred. This allowed for a comparison of the observed vaccination uptake for the vaccines during the pandemic period with the projected uptake in the hypothetical absence of an interruption.

The effect of the interruption was assessed by comparing observed trends before and after the interruption, with predicted trends after the interruption using 95% confidence intervals (CIs).

Secondary analysis was carried out on five counties to assess the effect of the pandemic on the two selected vaccines. Analyses were stratified according to five counties selected based on their climatic conditions and socio-economic status. Nairobi and Mombasa represent the densely populated urban city counties, while Kilifi and Kwale represent more rural less-populated coastal areas. Mandera includes arid counties in Northern and North-Eastern Kenya.

## 3. Results

**Figure 1:**
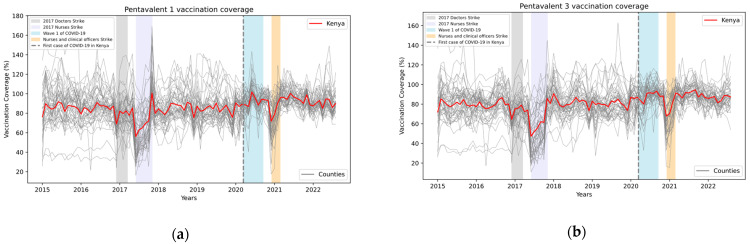
Monthly vaccination coverage (%) with pentavalent first (**a**) and third (**b**) dose in Kenya, overall, and for all counties in Kenya, with some events highlighted. Source: Data from KHIS; computations and design by authors.

Vaccine coverage rates in Kenya for the first and third dose of the pentavalent vaccine are illustrated in Figure 1.

**Figure 2:**
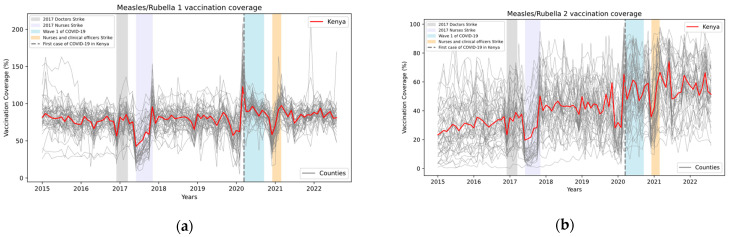
Monthly vaccination coverage (%) with measles/rubella first (**a**) and second (**b**) dose in Kenya, overall, and for all counties in Kenya, with some events highlighted. Source: Data from KHIS; computations and design by authors.

The first and the third dose of the pentavalent vaccine were selected for analysis due to their importance in assessing access (first dose) and continuity of use of facilities (third dose) by both UNICEF and WHO [21,57]. Rates for the first and second dose of the measles/rubella vaccine are shown in Figure 2.

**Figure 3:**
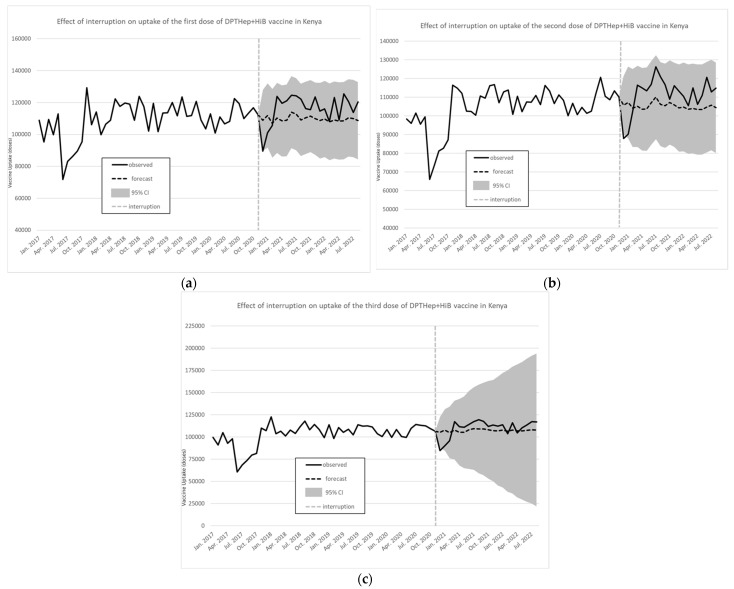
Effect of interruption on uptake of the first (**a**), second (**b**), and third (**c**) dose of pentavalent vaccine in Kenya. Source: Data from KHIS; computations and design by authors.

For the pentavalent vaccine, Figure 3a–c illustrates the monthly number of doses and forecasts in the absence of an interruption, with the shaded area representing the 95% CI of the forecasted values.

A visual examination of the trend suggested two main interruptions, respectively, in June 2017, likely due to the strike of healthcare workers, and in December 2020 during the COVID-19 pandemic. At the beginning of the interruption in December 2020, there was a decrease (step) of 8336 monthly vaccinations (95% CI −24,507 to 7833), while the slope (ramp) change further represented an increase of 889 monthly vaccinations (95% CI −287 to 2065). Figure 3b,c shows the results for the second and third doses of the pentavalent vaccine. For the third dose of pentavalent, at the beginning of the interruption in December 2020, there was a significant immediate decrease of 20,849 monthly vaccinations (95% CI −35,299 to −6398), while the slope change further represented an increase of 1426 monthly vaccinations (95% CI −2325 to 5177).

**Figure 4:**
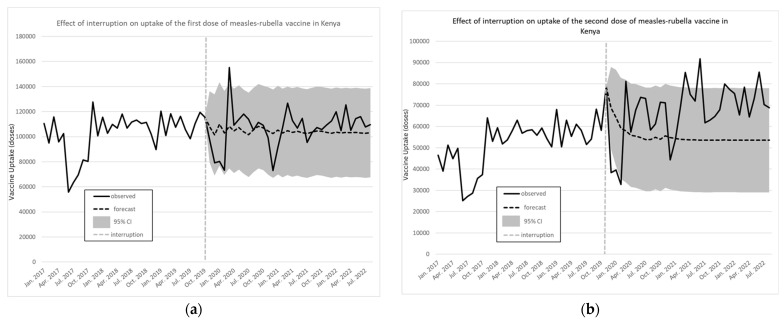
Effect of interruption on uptake of the first (**a**) and second (**b**) dose of measles/rubella vaccine in Kenya. Source: Data from KHIS; computations and design by authors.

Similar results are presented in Figure 4a,b for the monthly number of doses for the first and second uptake of the measles/rubella vaccine, respectively. For the interruption in December 2020 of the first dose of the measles/rubella vaccine, there was an immediate decrease of 6609 monthly vaccinations for the first dose (95% CI −24,184 to 10,966), while the slope change further represented an increase of 885 monthly vaccinations (95% CI −411 to 2181). For the second dose, the ARIMA model provided an immediate increase of 762 monthly vaccinations (95% CI −14,205 to 15,729), while the slope change further represented an increase of 361 monthly vaccinations (95% CI −888 to 1610).

**Figure 5:**
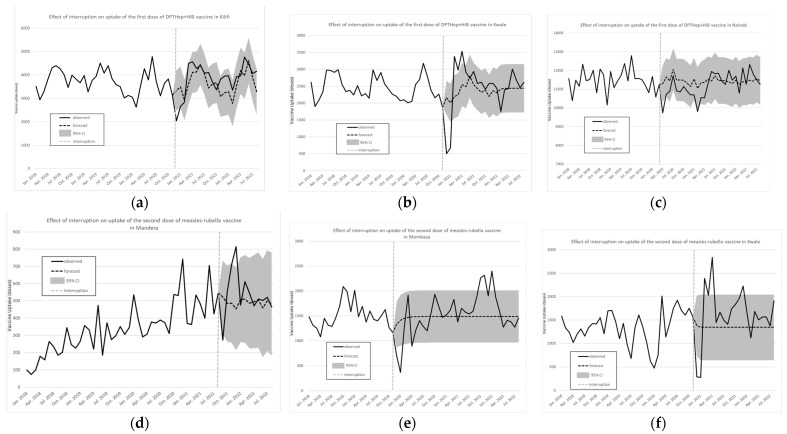
Effect of interruption on uptake in the first dose of the pentavalent vaccine in Kilifi County (**a**), Kwale county (**b**), and Nairobi County (**c**). Effect of interruption on uptake of the second dose of the measles/rubella vaccine is shown for Mandera county (**d**), Mombasa county (**e**), and Kwale county (**f**). Source: Data from KHIS; computations and design by authors.

The dose uptake in counties is shown in Figure 5a–f. Consistent with the patterns observed for the country overall, uptake for both vaccines and all doses from the rural counties of Kilifi and Kwale dropped in December 2020 and later went back to values similar to or higher than those prior to the interruption. Mandera, one of the arid counties, experienced only a slight drop in March uptake of the pentavalent vaccine and no subsequent drops. The third dose of the vaccine did not experience any notable variations, accounting for seasonal effects. In addition, for the measle/rubella vaccine, a surge was observed for both doses in March 2020. This corresponded to a rise in immunization following a two month stock-out between November 2019 and January 2020 [28]. In Nairobi, Kenya’s capital city, a drop was observed in April 2020 for all three doses of pentavalent, while a drop was observed in December 2020 for the first dose of measles/rubella vaccine.

There was no clear pattern for the monthly vaccine uptake in Mandera and Mombasa counties. In Mandera county, no significant drop was observed for the first dose of the measles/rubella vaccine. A significant drop was observed in July 2021, but no specific event was found at that time. A similar drop, at the same time, was observed in Nairobi County for the second dose of the measles/rubella vaccine. Neither Nairobi nor Mombasa counties had a significant drop in uptake of the first dose of the measles/rubella vaccine. Mombasa County had a significant drop just before the pandemic began, in December 2019, and subsequent rates remained normal, accounting for seasonal effects.

The immediate (step) and long-term (ramp) changes are summarized in Table 1. Only the third dose of the pentavalent vaccine had a significant immediate decrease in the number of doses.

## 4. Discussion

There was no major impact of public health measures instituted during the pandemic on the uptake of the pentavalent and measles/rubella vaccine in Kenya. The major drop seen across all three doses of pentavalent vaccine and the two doses of measles/rubella vaccine was in December 2020, coinciding with the beginning of a 70-day joint strike by nurses and clinical officers in Kenya. This strike was initiated to protest against the absence of personal protective equipment, the withdrawal of tax reliefs, and the need for COVID-19 vaccines. Normal uptake of vaccination resumed once the strike ended in February 2021.

This analysis revealed a major impact of healthcare worker strike on the uptake of routine immunization. Policy measures should be put in place to ensure the continuity of immunization services during such industrial unrest to ensure that gains made in vaccination coverage are not lost. This analysis also revealed the impact of COVID-19 on routine immunization, showing the initial slowdown in uptake of vaccination in general was more pronounced for older infants, who usually receive the measles containing vaccine at 9 months, than for younger infants, who receive the pentavalent vaccine at 6 to 14 weeks [58].

Uptake was also more greatly reduced in rural areas than in urban areas. COVID-19-related interventions or disruptions, such as healthcare worker strikes, affected routine immunization in Kenya, and recovery was slow, taking two to four months after the fact to reach pre-COVID-19 levels.

Results from similar studies in Brazil assessing the impact of COVID-19 using interrupted time series on under-one vaccination and against Meningococcal C infection were varied. While the first study reported no impact of the pandemic on vaccination, the second study reported a statistically significant negative impact of the pandemic on vaccination against meningococcal C infection in the north and south regions, as well as eleven Brazilian states [59,60]. Another study in Canada assessed the impact of vaccine mandate announcement of COVID-19 vaccine uptake, and found that vaccine mandate announcements could have increased vaccine uptake [61].

Closer to Kenya, a study in a hospital in Uganda analyzed whether COVID-19 affected maternal, neonatal, and child health services. It reported a reduction in immunization clinic attendance during the lockdown due to the pandemic [62]. A mixed methods study, also utilizing interrupted time series in Kenya, assessed the impact of the initial pandemic-related interventions on, among other things, the coverage of the third dose of the pentavalent vaccine and measles vaccine coverage. It reported a non-statistically significant increase in coverage of the two vaccines during the pandemic, but attributed the rise in uptake of measles vaccine to catch-up campaign to cover for supply chain shortfalls [28].

Our study had limitations. Firstly, we could not address all childhood vaccines of the routine immunization program, such as HPV, owing to the short period it had been in use, which would have constrained analysis using ARIMA modelling. Another limitation was the lack of a control group for comparison of trends. In the case of COVID-19, the pandemic affected the whole world, and therefore there was no comparison group of “unexposed” controls. It is therefore not possible to conclude a causal association between the COVID-19 pandemic and the observed trends in vaccination rates. Furthermore, assessment of multiple interruptions occurring in sequence was not possible with the methods utilized.

As interrupted time series analysis is a widely accepted method to assess the impact of policy interventions or major events in the population, this study has demonstrated that up-to-date vaccination data are available in Kenya. Time series analyses should therefore be conducted systematically after any major event to assess its impact and inform rapid corrective action. Time series analyses can also be conducted routinely semi-annually to monitor progress and detect any underlying variations in trends by region.

## 5. Conclusions

The COVID-19 pandemic had direct and indirect effects on routine immunization uptake. Our study demonstrates an indirect effect of the pandemic that could have caused a direct reduction in uptake in childhood vaccines in Kenya. Specifically, a statistically significant drop in the number of doses for the third dose of the pentavalent vaccine was noted. These effects were likely due to the healthcare worker strike during the pandemic. The pentavalent vaccine, administered earlier in life, showed to be less affected than the measles–rubella vaccine, which is given later in life. Further research using epidemiologic approaches is necessary to ascertain the effect of the COVID-19 pandemic on routine immunization and to inform corrective actions in future pandemics.

## Figures and Tables

**Table 1:** Impact of the December 2020 interruption on vaccination using interrupted autoregressive integrated moving average models.

	First Month of the Interruption			Immediate Change ^a^ (95% CI)	Slope Change ^b^ (95% CI)
Vaccine	Observed	Forecasted (95% CI)	Relative Difference (%)		
Pentavalent, 1st dose	89,590	108,939 (89,831 to 128,047)	−21·6	−8336 (−24,507 to 7833)	889 (−287 to 2065)
Pentavalent, 2nd dose	87,953	105,661 (90,107 to 121,216)	−20·1	−12,212 (−27,872 to 3448)	1106 (−162 to 2374)
Pentavalent, 3rd dose	84,616	105,353 (87,773 to 122,934)	−24·5	−20,848 (−35,299 to -6397)	1426 (−2325 to 5177)
Measles/rubella, 1st dose	73,023	92,965 (61,106 to 124,824)	−27·3	−6609 (−24,184 to 10,966)	885 (−411 to 2181)
Measles/rubella, 2nd dose	44,335	60,138 (37,274 to 83,003)	−35·6	762 (−14,205 to 15,729)	361 (−888 to 1610)

^a^ Step intervention function. ^b^ Ramp intervention function; CI: Confidence interval.

## Data Availability

Restrictions apply to the availability of these data. Data were obtained from the Kenya Health Information System and are available from the Ministry of Health, Kenya, with the permission of the Ministry of Health.

## Appendix A

Timeline of events in Kenya

5 December 2016–14 March 2017: 100 days of Doctor’s strike.

5 June 2017–3 November 2017: 150 days of nurse’s strike.

13 March 2020: First case of coronavirus in Kenya confirmed.

20 March 2020: Closing of all schools and higher learning institutions.

25 March 2020: 7 pm–5 am curfew announced in Kenya.

27 March 2020: National dusk-to-dawn curfew imposed.

6 April–7 July 2020: Cessation of movement in and out of the Nairobi Metropolitan Area.

8 April–7 July 2020: Cessation of movement in and out of Mombasa County.

8 April–7 June 2020: Cessation of movement in and out of Kilifi and Kwale counties.

22 April–7 July 2020: Cessation of movement in and out of Mandera county.

6 May–6 June 2020: Cessation of movement in and out of Eastleigh in Nairobi and Old Town area in Mombasa. Closure of markets, restaurants, and eateries within those areas.

On 17 June 2020, the Government of Kenya announced further restrictions in response to the worsening of the COVID-19 pandemic. Restrictions were focused on 13 counties each declared to be a “hotspot zone”, specifically Busia, Vihiga, Kisii, Nyamira, Kakamega, Trans Nzoia, Bungoma, Kericho, Bomet, Siaya, Kisumu, Homa Bay, and Migori.

These restrictions in the 13 counties included the following:

Public gatherings were suspended until further notice.

The curfew in the 13 counties was restricted from 7 pm to 4 am. Other counties maintained the existing curfew from 10 pm to 4 am.

Except for essential and emergency services, movement between the hotspot zone and the rest of the country was strongly discouraged.

Non-food and livestock markets was suspended for 30 days.

Employers were advised to allow employees to work from home.

All physical and worship gatherings were suspended for 30 days.

Hospitals restricted visitors to one individual per patient.

All funerals were held within 72 h after the confirmation of death, and no more than 50 people could attend.

Weddings were restricted to 30 people.

7 December 2020–23 February 2021: 70 day strike comprising nurses and clinical officers.

20 October 2021: The curfew was lifted.

11 March 2022: The government lifted all remaining COVID-19 restrictions, including a ban on large indoor gatherings such as religious services and a requirement to present a negative COVID-19 test for arriving air passengers.

29 May–28 July 2022: Campaign season for the 2022 general elections.

9 August 2022: Kenya general elections conducted.
